# Role of Mir-155 in Controlling HIF-1α Level and Promoting Endothelial Cell Maturation

**DOI:** 10.1038/srep35316

**Published:** 2016-10-12

**Authors:** Deguang Yang, Jinhong Wang, Meng Xiao, Tao Zhou, Xu Shi

**Affiliations:** 1 Department of Cardiology, the Third Affiliated Hospital of Southern Medical University, Guangzhou, 510000, China; 2 Department of Respiration, the Third Affiliated Hospital of Southern Medical University, Guangzhou, 510000, China; 3 Department of Nursing, the Third Affiliated Hospital of Southern Medical University, Guangzhou, 510000, China; 4 Central Laboratory, the First Hospital of Jilin University, Changchun, 130032, China

## Abstract

Stem-cell-based therapy for cardiovascular disease, especially ischemic heart disease (IHD), is a promising approach to facilitating neovascularization through the migration of stem cells to the ischemic site and their subsequent differentiation into endothelial cells (ECs). Hypoxia is a chief feature of IHD and the stem cell niche. However, whether hypoxia promotes stem cell differentiation into ECs or causes them to retain their stemness is controversial. Here, the differentiation of pluripotent stem cells (iPSCs) into endothelial cells (ECs) was induced under hypoxia. Though the angiogenic capability and angiogenesis-related autocrine/paracrine factors of the ECs were improved under hypoxia, the level of hypoxia inducible factor 1α (HIF-1α) was nonetheless found to be restricted along with the EC differentiation. The down-regulation of HIF-1α was found to have been caused by VEGF-induced microRNA-155 (miR-155). Moreover, miR-155 was also found to enhance the angiogenic capability of induced ECs by targeting E2F2 transcription factor. Hence, miR-155 not only contributes to controlling HIF-1α expression under hypoxia but also promotes angiogenesis, which is a key feature of mature ECs. Revealing the real role of hypoxia and clarifying the function of miR-155 in EC differentiation may facilitate improvement of angiogenic gene- and stem-cell-based therapies for ischemic heart disease.

Cardiovascular disease is the leading cause of morbidity and mortality worldwide, causing 17.3 million deaths in 2013, an increase from 12.3 million in 1990. In particular, ischemic heart disease (IHD)—which mainly refers to coronary artery disease (CAD) such as angina and myocardial infarction—is the most common cause of death globally, contributing to 8.14 million premature deaths in 2013^1^,^2^.

IHD treatment is directed toward re-establishment of blood flow to the ischemic area, and angiogenesis is key to promoting vascular network reconstruction. Endothelial cells (ECs) lining blood vessels control vessel function, regulating both vascular tone and neovascularization. Injury or dysfunction of ECs has been shown to contribute to IHD^3^,^4^,^5^. An innovative option for IHD treatment involves the transplantation of endothelial progenitor cells (EPCs); however, EPCs in peripheral blood are limited, complicating the clinical application of this technique.

Recently, stem cell-based therapy has emerged as a potential approach for treating IHD. Circulating stem and progenitor cells, induced pluripotent stem cells (iPSCs), resident cardiac stem cells, and mesenchymal stem cells (MSCs) have the potential to promote neovascularization by migrating to the ischemic site and differentiating into ECs^6^,^7^,^8^. Although the potential of using stem cells as a source of ECs has been proved, the mechanism underlying the process of EC differentiation is not yet clear.

It is generally known that hypoxia is a major characteristic of the microenvironment in ischemic tissues. Consequently, once stem cells migrate to an ischemic site, a series of cellular functions—especially those associated with angiogenesis—change in response to hypoxia. Hypoxia-inducible factor-1α (HIF-1α), a master effector of hypoxia, regulates many genes involved in cellular proliferation, migration, energy metabolism, angiogenesis, and apoptosis^9^,^10^. Numerous studies indicate that hypoxic modulation of cell function can be mediated by microRNAs, which are single-stranded noncoding RNAs of 22–25 nucleotides. MicroRNAs can induce the degradation of specific genes by targeting and combining with the 3′-UTR of mRNA^11^,^12^. HIF-1α is reported to up-regulate miR-27, miR-155, miR-199 and miR-210, and to down-regulate miR-221, miR-222 and miR-320^12^,^13^. Several studies also suggest that some microRNAs, such as miR-155, can control HIF-1α, forming a HIF-1α-miR-155 negative feedback loop to maintain the oxygen homeostasis^14^,^15^. Even so, the manner in which hypoxia influences EC differentiation and function (such as angiogenic capability) is not yet clear.

In the present study, we induced iPSCs to differentiate into ECs under hypoxia or normoxia *in vitro.* Then, we investigated the effects of hypoxia on EC differentiation and angiogenesis. Results showed that miR-155 is a key promoter for EC maturation rather than HIF-1α. The high level of miR-155 induced by VEGF was found to mediate angiogenesis by targeting E2F2 transcription factor. Determining the role of hypoxia during EC differentiating and clarifying the function of miR-155 in this process would be of great significance to improving angiogenic gene- and stem-cell-based therapies for ischemic heart disease.

## Results

### Differentiation of iPSCs into ECs *in vitro*

Generally, induction of EC differentiation is driven by the generation of embryoid bodies (EBs) and then treatment with stimuli such as Activin A, VEGF and BMP-4 allow progression. Subsequently, specific cells positive for KDR, CD31, or VE-cadherin, which are endothelial cell markers, are sorted and continually cultured with VEGF^16^,^17^,^18^,^19^. Recently, Chen *et al.*^20^ reported that differentiated cells with the typical morphology and immunophenotype of ECs could be obtained within 8 days via stimulation with VEGF alone.

**Figure 1:**
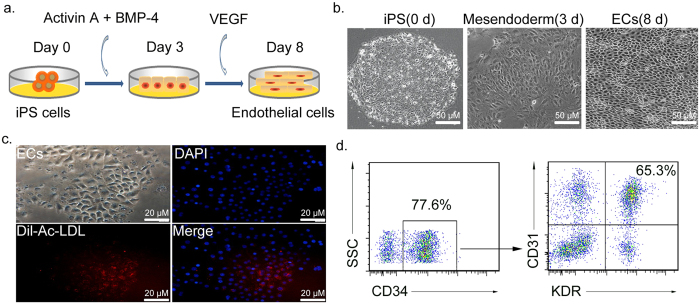
Strategy for iPS differentiation into ECs *in vitro*. (**a**) Schematic flow chart of EC differentiation process. (**b**) Morphological changes of iPSCs at day 0, 3 and 8. Scale bars = 50 μm. (**c**) Uptake of Dil-Ac-LDL representing endothelial function (red). Scale bars = 20 μm. (**d**) Flow cytometric analyses of EC markers. Differentiated cells (1 × 10^5^) were incubated with primary antibodies against CD34, and then CD31 and KDR double-positive cells in CD34^+^ population were identified as induced ECs. % indicates the fraction of cells stained positive.

In view of induction efficiency and convenience of observation, an induction protocol that comprehensively applied the advantages of the two methods mentioned above was used. Briefly, Activin A and BMP-4 were initially used to produce meso-endodermal cells, and then VEGF was added to trigger the formation of ECs (Fig. 1a). As shown in Fig. 1b, cells with EC morphology were successfully induced. These induced cells were also able to take up human Dil-Ac-LDL (Fig. 1c), which is a hallmark of ECs. Dil-Ac-LDL does not bind to LDL receptors but rather to scavenger receptors expressed on vascular ECs, macrophages, and EPCs^21^.

Generally, CD34 is a common marker of ECs and hematopoietic stem cell (HSCs), and CD31 is normally found on ECs. KDR (vascular endothelial growth factor receptor 2, VEGFR-2) can often be found on HSCs at early differentiation stage, but it gradually declines along with HSC differentiation while increases in mature ECs. Therefore, CD31^+^KDR^+^ cells in CD34^+^ subset are commonly recognized as ECs^22^,^23^. As shown in Fig. 1d, the induced cells were immunophenotypically similar to mature ECs (positive for CD31^+^KDR^+^ (64.8% + 4.7%) in CD34^+^ cells). These data indicated that ECs had been successfully produced from iPSCs.

### Angiogenic capability of induced ECs is improved under hypoxia

Hypoxia is a major characteristic of the microenvironment in ischemic tissues, so it is closely associated with EC differentiation. White *et al.*^19^ used hypoxic condition (5% CO_2_, 5% O_2_, 90% N_2_) to induce ECs from iPSCs. Fasanaro *et al.*^24^ reported that ECs respond to hypoxia via the proangiogenic microRNA, miR-210. However, it has also been reported that hypoxia in the stem cell niche causes the cells to retain their pluripotency. Thus, the effect of hypoxia on stem cell differentiation is subject to complex and comprehensive modulation.

**Figure 2:**
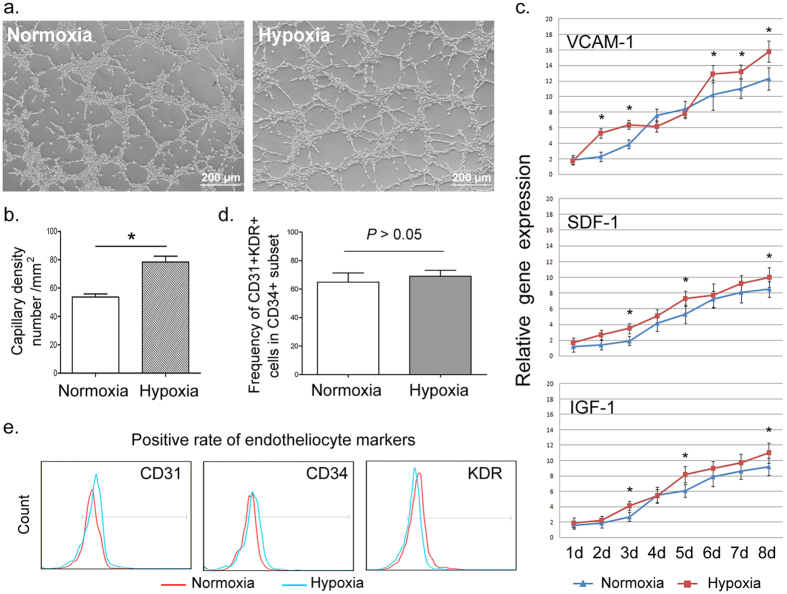
Effects of hypoxia on EC differentiation. (**a,b**) Representative photomicrograph and quantification of microtubule formation under hypoxia and normoxia. Differentiated cells under hypoxia had significantly enhanced vascular tube formation. Scale bars = 200 μm. (**c**) Expression of angiogenesis-associated factors (VCAM-1, SDF-1 and IGF-1) during EC differentiation under hypoxia and normoxia. Hypoxia generally improved these angiogenic factors. (**d,e**) Flow cytometry analysis was performed to compare the positive rate of induced ECs (CD34^+^CD31^+^KDR^+^) and each single endothelial marker between hypoxic and normoxic conditions. All data are represented as means ± SD. *P < 0.05 vs. normoxia group.

Here we induced the differentiation of iPSCs into ECs under hypoxic and normoxic conditions respectively. As shown in Fig. 2a,b, with low O_2_, induced ECs were better able to form microtubules, which is a widely used indicator to evaluate the angiogenic capability of functional mature ECs. Lee *et al.*^25^ indicated that pretreatment with hypoxia enhanced differentiation of EBs into meso-endodermal cells by up-regulating VEGF expression, and this process was closely associated with the autocrine/paracrine capability of EBs. Therefore, we compared the level of angiogenesis-related autocrine/paracrine factors such as VCAM-1, SDF-1 and IGF-1^26^,^27^,^28^ under hypoxic and normoxic condition respectively. Though several other cytokines, such as G-CSF, GM-CSF and IL-1 also promote angiogenesis^29^,^30^,^31^, they are closely related to immune cells. As stated in previous reports, VCAM-1 reflects the activation of ECs^32^, IGF-1promotes angiogenesis by stabilizing neovessels^33^ and SDF-1directly promotes angiogenesis by recruiting EPCs from bone marrow^34^. Hence, VCAM-1, SDF-1 and IGF-1 are here considered more closely associated with the development of endotheliocyte itself, and they can reflect the activation and maturation of ECs. As shown in Fig. 2c, VCAM-1, SDF-1 and IGF-1 were all highly expressed under hypoxia even at the early differentiation stage, indicating hypoxia might enhance the angiogenic ability of ECs.

Unlike the improved angiogenesis, the positive rate of induced mature ECs (CD31^+^KDR^+^ cells in CD 34^+^ subset) and each single endothelial marker showed nearly no significant differences between hypoxia and normoxia (Fig. 2d,e). That indicated that hypoxia might promote the secretion of angiogenic cytokines in iPSCs rather than increasing the differentiation efficiency of iPSCs.

### Dynamic expression of miR-155 and HIF-1α during EC differentiation

**Figure 3:**
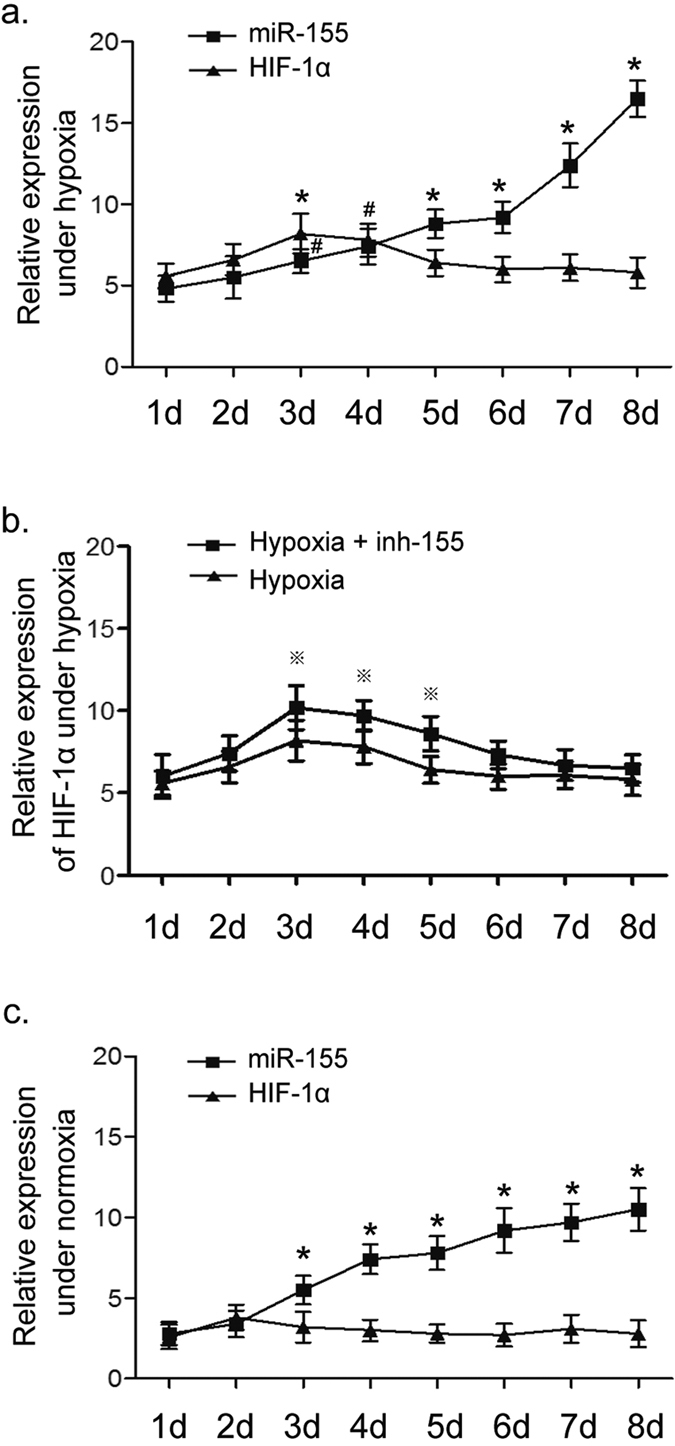
Dynamic changes in HIF-1α and miR-155 expression during EC differentiation under hypoxia or normoxia. Time profiles of HIF-1α and miR-155 gene expression were measured with quantitative real-time PCR. (**a)** The expression of HIF-1α and miR-155 under hypoxia. MiR-155 gradually increased as EC differentiation continued, while HIF-1αraised first and then decreased. *P < 0.05 vs. day 1 as for miR-155 level; ^#^*P* < 0.05 vs. day 1 as for HIF-1α level. (**b**) Comparison of HIF-1α levels between the hypoxia group and hypoxia plus miR-155 inhibitor (inh-155) group. Inhibiting miR-155 contributed to a higher level of HIF-1α first, and then HIF-1α declined to a level similar to that in hypoxia group without inh-155. ^※^*P* < 0.05 vs. hypoxia group. (**c**) The expression of HIF-1α and miR-155 under normoxia. MiR-155 began to increase significantly once the cells were stimulated with VEGF (day 3). Meanwhile, differentiated cells also expressed HIF-1αregardless of normoxia, although it was at a low and stable level. *P < 0.05 vs. day 1 as for miR-155. All data are represented as means ± SD.

Because hypoxia enhanced the angiogenic ability of ECs^35^,^36^, we then investigated whether it was directly mediated by HIF-1α, a master hypoxia effector. Figure 3a showed that, with low O_2_, the expression of HIF-1α gradually increased from day 0 to day 3. However, this trend was sustained for only 72 h. After day 4, HIF-1α levels gradually declined. These data indicated that HIF-1α was negatively regulated by certain factors in the later stage of differentiation even under prolonged hypoxia, so we speculated that avoiding high level of HIF-1α might be essential to EC differentiation.

To explore the decline of HIF-1α, the current work focused on miR-155, a negative regulator of HIF-1α. MiR-155 is initially reported to be up-regulated by HIF-1α, and recently, it has been proven to inhibit HIF-1α transcription by targeting its mRNA 3′-UTR^14^,^15^. Thus, the HIF-1α−miR-155 negative feedback loop may help keepHIF-1α expression at a reasonable level during EC differentiation. As shown in Fig. 3a, miR-155 level was obviously higher during the first 3 days under hypoxia. However, unlike the subsequent decline in HIF-1α, miR-155 expression was continuously increased over the following 5 days (Fig. 3a). These results might explain the decline in HIF-1α from day 4 to day 8, and furthermore, they also showed thatmiR-155 is disconnected from HIF-1α regulation, and the HIF-1α−miR-155 negative feedback loop had been broken in the later differentiation. So, there must be a positive regulator to promote miR-155 expression during EC differentiation.

To confirm the regulation of HIF-1α by miR-155, HIF-1α expression was detected in a hypoxia group supplemented with a chemical synthetic miR-155 inhibitor (inh-155). As shown in Fig. 3b, the level of HIF-1α was initially increased after inhibition ofmiR-155, but it eventually declined to a level similar to that in single hypoxia group at the end of the induction process. The results suggested that miR-155 indeed down-regulated HIF-1α expression in EC differentiation under hypoxia, but there were other mechanisms underlying the maintenance of HIF-1α level.

To clarify whether HIF-1α expression was necessary for induction of ECs, we further detected the expression of miR-155 and HIF-1α using the same induction protocol under normoxia. The level of miR-155 gradually increased along with the treatment of VEGF. For this reason, VEGF is here considered a positive regulator of miR-155, and the high level of miR-155 might promote EC differentiation. In addition, the induced ECs continued to express HIF-1α under normoxia at low but stable levels (Fig. 3c). Therefore, we hypothesize that there exist precise mechanisms that keep HIF-1α expression at a reasonable level during EC differentiation regardless of whether conditions are hypoxic or normoxic condition.

### VEGF directly induces expression of miR-155 through the ERK signaling pathway

Because miR-155 level increased after VEGF stimulation under both hypoxia and normoxia, VEGF was here considered to directly promote miR-155 expression. As a type of mature ECs, human umbilical vein endothelial cells (HUVECs) are initially isolated from the umbilical cord vein and commonly marked by CD31, CD34 and KDR. Though the induced cells are also mature ECs, their characteristics are not exactly the same as HUVECs’. However, these two cell types shared main features of mature ECs such as angiogenesis ability, Dil-Ac-LDL uptake and cell surface markers (CD31, CD34 and KDR). Hence, HUVECs were chosen to be a cell model for study of the role of miR-155 in the process of microtubule formation and the regulation of miR-155 by VEGF.

**Figure 4:**
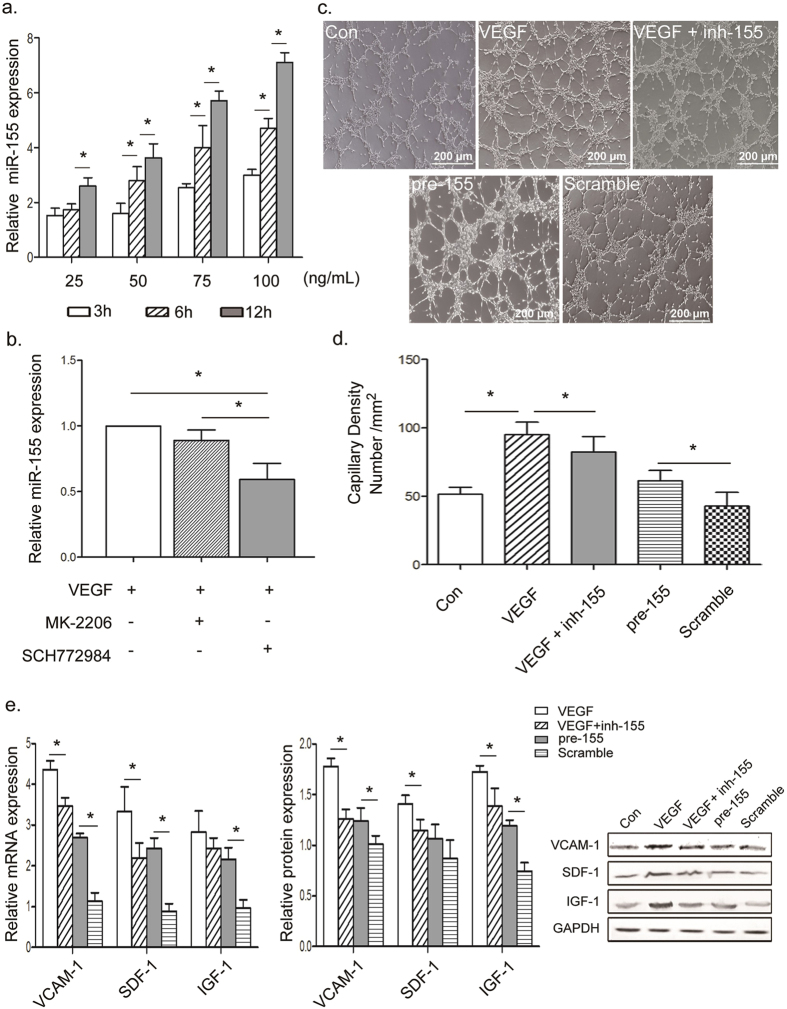
Prolonged VEGF stimulation increased miR-155 by activating the ERK signaling pathway. (**a**) miR-155 expression was regulated by VEGF in a time- and dose-dependent manner. (**b**) After stimulation with 50 ng/mL VEGF for 12 h, miR-155 significantly decreased in the group treated with ERK inhibitor compared with those treated with AKT inhibitor. That indicated that VEGF modulated miR-155 via the ERK signaling pathway. (**c,d**) Representative photomicrograph and quantification of microtubule formation, depicting effects of VEGF and miR-155 in HUVECs. Angiogenic function of HUVECs was proved to be positively correlated miR-155. Scale bars = 200 μm. (**e,f**) qPCR and Western blot analysis for the expression of VCAM-1, SDF-1 and IGF-1 in VEGF or pre-155 treatment groups in HUVECs. Pre-155 alone was found to render levels of proangiogenic cytokines, while inh-155 partially reversed VEGF-induced angiogenesis factors. All data are means ± SD. *P < 0.05.

Initially, to confirm whether VEGF promotes miR-155, HUVECs were stimulated with VEGF and miR-155 expression was detected. As shown in Fig. 4a, miR-155 was significantly up-regulated by VEGF in a time- and dose-dependent manner. A series of studies shows that VEGF induces angiogenesis by stimulating the proliferation, migration and sprouting of ECs, and these effects are associated with the activation of VEGF downstream signaling, including AKT and ERK pathways^37^. The role of VEGF/AKT and VEGF/ERK signaling in the regulation of miR-155 was explored further. In the presence of 50 ng/mL VEGF, HUVECs were treated with an AKT inhibitor MK-2206 (0.5 μM) and an ERK1/2 inhibitor SCH772984 (1 μM) respectively for 12 h. As shown in Fig. 4b, the expression of miR-155 was primarily suppressed in the group with ERK inhibitor. These results demonstrated that VEGF could promote miR-155 expression by activating VEGF/ERK signaling pathway.

Subsequently, noting that VEGF-induced miR-155 must benefit the differentiation of EC, whether miR-155 is a major mediator of angiogenesis was investigated further. Specifically, HUVECs were treated with an expression vector containing miR-155-5p precursor clone (pre-155, with the mature sequence: uuaaugcuaaucgugauaggggu) and a chemical synthetic miR-155 inhibitor (inh-155). As shown in Fig. 4c,d, VEGF significantly enhanced the angiogenic ability of HUVECs. Meanwhile, in the presence of VEGF, the ability of HUVEC to form microtubules was inhibited in the group with inh-155. Single pre-155 also seemed to be able to promote microtubule formation. In addition, the levels of angiogenesis-related factors (VCAM-1, SDF-1 and IGF-1) were visibly up-regulated in pre-155 group and down-regulated in inh-155 group (Fig. 4e). It is here concluded that the activation of VEGF/ERK signaling pathway promoted miR-155 expression, leading to the improvement of EC angiogenic ability. Therefore, VEGF-induced miR-155 could positively regulate EC differentiation by improving the functional maturation of induced ECs.

### MiR-155 improves endothelial angiogenesis via E2F2 transcription factor

After confirming the effect of miR-155 on promoting endothelial angiogenesis, we further explored the underlying mechanism of miR-155by scanning potential targets using TargetScan (http://www.targetscan.org) and MiRBase (http://www.mirbase.org). In view of the data gathered from these databases, the investigation focused on the transcription factor E2F2, which belongs to the E2F family that participates in cell proliferation, apoptosis and development^38^. E2F2 has been thoroughly studied and found to control diverse physiological and pathological activity in ECs. Zhou *et al.*^39^ reported that capillary density and proliferation of ECs could be enhanced by the loss of E2F2 expression. E2F2 also functions in stem cells. Suzuki *et al.*^40^ demonstrated knockdown of E2F2 preserved stemness of human embryonic stem cells, and Wu *et al.*^41^ showed that miR-125b could regulate the proliferation of glioblastoma stem cells by targeting E2F2.

**Figure 5:**
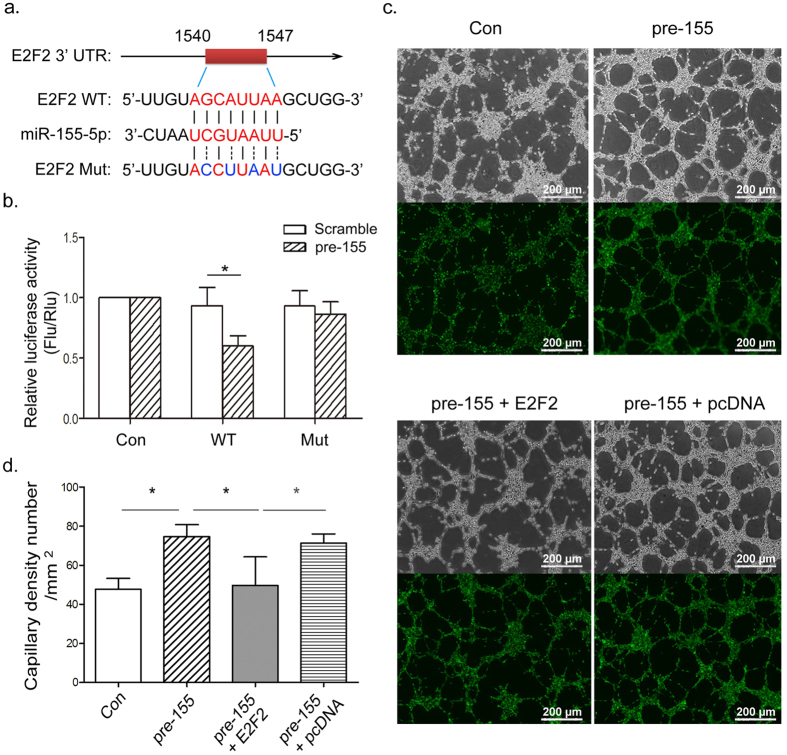
MiR-155 enhanced microtubule formation by suppressing E2F2 transcription factor. (**a**) Sequence alignment of the human miR-155-5p mature sequence with the binding sites of human E2F2 3′-UTR wild-type (WT) and mutant (Mut). (**b**) Changes in luciferase activity of the pGL3-E2F2-3′-UTR WT and Mut after co-transfection with pre-155-5p. (**c,d**) Representative microtubule formation of HUVECs after transfection with pre-155, pre-155 combined with E2F2 expression plasmid (pcDNA-E2F2) and empty pcDNA control. MiR-155 partially improved microtubule formation by inducing E2F2 degradation. Scale bars = 200 μm. All data are means ± SD. *P < 0.05.

Previous reports have shown E2F2 to be a verified target of miR-155 in colorectal carcinoma and renal carcinoma cells^42^,^43^. Thus, we explored whether miR-155 could facilitate endothelial angiogenesis by inhibiting E2F2 transcription in HUVECs. A luciferase reporter vector containing a segment of E2F2 3′-UTR was constructed and subsequently co-transfected into HUVECs in combination with a pre-155 plasmid. Data indicated that miR-155 could bind directly to the 3′-UTR region of E2F2 mRNA (Fig. 5a,b). HUVECs were then treated with pre-155 and an E2F2 expression vector (pcDNA-E2F2) and the results proved microtubule formation could be improved by pre-155 and suppressed by exogenous E2F2 expression (Fig. 5c,d). In this way, VEGF-induced miR-155 might improve endothelial angiogenesis directly by suppressing E2F2.

## Discussion

Although techniques for differentiating stem cells into other specific types of cells are widely utilized today, the underlying regulatory mechanisms remain to be elucidated. Oxygen content in the cellular microenvironment influences cell proliferation, function, and differentiation. Hypoxia is known to be important to stem cells, but whether hypoxia contributes to pluripotency or differentiation remains controversial. Hypoxia has been shown to maintain the pluripotency of human hESCs and preserve expression of NANOG and SOX2 in adipose-derived stem cells^44^,^45^. However, other studies indicate that transient exposure to hypoxia enhances endoderm formation^46^ and induces differentiation of hESCs into cardiomyocytes^47^ and chondrocytes^48^.

In this study, iPSCs were induced to differentiate into ECs under hypoxia and normoxia. Results showed that induced ECs could better form microtubules and secrete more angiogenesis-related cytokines under hypoxia than under normoxia, but this was not directly mediated by the major hypoxia effector HIF-1α because it declined in the later induction even under prolonged hypoxic condition. To investigate the down-regulation of HIF-1α, we detected its negative regulator miR-155, and found miR-155 level gradually increased throughout the induction process under hypoxia. That might be the reason for declined HIF-1α level. However, it is still unclear which factor promotes miR-155 expression.

By analyzing the time traces of the expression of miR-155, we found its level began to increase significantly just after the cells were stimulated with VEGF. It was then proven that VEGF could directly promote miR-155 expression through VEGF/ERK signaling pathway, and the increased miR-155 was able to improve EC angiogenic ability by suppressing E2F2, a common pro-angiogenic factor. In this way, VEGF-induced miR-155 contributes to the functional maturation of induced ECs, indicating that miR-155 positively regulates EC differentiation.

One interesting thing in this induction process was that the expression of HIF-1α was transiently up-regulated within the first 72 h under hypoxia and that prolonged hypoxia did not stabilize HIF-1α. VEGF-induced miR-155 were here considered to mediate the decrease of HIF-1α under hypoxia, but after inhibiting miR-155, the level of HIF-1α eventually declined to a level similar to that without miR-155 inhibitor. Accordingly, avoiding a high level of HIF-1α and keeping HIF-1α expression at a low and stable level may be necessary in EC differentiation.

Fotia *et al.*^45^ reported that hypoxia has a dual character, helping cells to maintain their stem cell characteristics in the absence of osteogenic stimuli but inducing differentiation in bone-like microenvironments. In view of these data, we hypothesize that hypoxia’s effect on EC differentiation may be divided into 2 stages: During the early stage, though exposure to low O_2_ concentration may tend to maintain stemness, hypoxia enhances the secretory function of iPSCs, which helps them to adapt to the changing microenvironment by secreting certain stress-related factors (including pro-angiogenic cytokines). During the later stage, VEGF/ERK signaling is persistently activated, resulting in a high level of miR-155 that not only suppresses HIF-1α-induced stemness but also improves EC angiogenic ability by inhibiting E2F2 transcription factor.

In conclusion, results showed that in the VEGF-triggered EC differentiation *in vitro*, the effect of HIF-1α on EC maturation is limited especially in the later induction process. Meanwhile, VEGF-induced miR-155 plays an important role in improving EC functions such as microtubule formation and secretion of angiopoiesis-associated autocrine/paracrine factors. However, the mechanism underlying the control of HIF-1α level during EC differentiation is still unclear and needs further exploration.

## Materials and Methods

### Cell culture and differentiation

The human iPS cell line UCS0730C11 was maintained on gamma-ray-irradiated mouse embryo fibroblasts (both from Sidansai, Shanghai, China) with Dulbecco’s modified Eagle’s medium/F-12 (DMEM/F-12) containing 20% knockout serum replacement (KSR), 1× nonessential amino acids, 2 mM L-glutamine, 0.1 mM β-mercaptoethanol, 20 ng/mL recombinant human fibroblast growth factor basic, and 1% penicillin-streptomycin (all from Gibco, CA, U.S.). iPS cells were cultured at 37 °C in a tri-gas incubator (3% O_2_, 5% CO_2_, and 92% N_2_; SANYO, Osaka, Japan) to establish the hypoxic cellular model. Human umbilical vein endothelial cells (HUVECs) initially phased from the American Type Culture Collection (ATCC) were kept in our laboratory and cultured with DMEM/F-12 medium supplemented with 10% fetal bovine serum (FBS), 2 mM L-glutamine, 20 ng/mL recombinant human epidermal growth factor (EGF), 0.1 mg/mL heparin, and 1% penicillin and streptomycin (all from Gibco).

To induce endothelial differentiation, iPS cells were seeded on type IV collagen (5 μg/mL, Stemcell Technologies, BC, Canada) in differentiation medium containing α-MEM medium, 10% FBS, 10 ng/mL basic fibroblast growth factor (bFGF), 0.05 mM β-mercaptoethanol, 1% penicillin and streptomycin (all from Gibco). During the first 2 days, iPSCs were supplemented with 6 ng/mL Activin A (PeproTech, NJ, U.S.) and 10 ng/mL BMP-4 (R&D, MN, U.S.). Then Activin A and BMP-4 were replaced with 50 ng/mL VEGF (PeproTech) for generating ECs from day 3 to day 8.

### Immunophenotypic identification of induced ECs

Induced ECs were digested with 2 U/mL Dispase (Stemcell Technologies, BC, Canada) and then collected for flow cytometry. Fluorescent labeled primary antibodies, PE Mouse Anti-Human CD31, FITC Mouse Anti-Human CD34, and APC Mouse Anti-Human KDR were used to label ECs^16^. Respective isotypes were used as negative controls. All antibodies were purchased from BD Pharmingen CA, U.S. Cells were analyzed by flow cytometry using a FACSCalibur (BD Biosciences, NJ, USA).

### Uptake of human Dil-labeled acetylated low-density lipoprotein (Dil-Ac-LDL)

The induced endothelial cells were incubated with 10 μg/ml Dil-Ac-LDL (Molecular Probes, CA, U.S.) for 4 h at 37 °C. To determine the amount of Dil-Ac-LDL, cells were washed twice with PBS and visualized via fluorescence microscopy (Olympus IX71, Olympus, Tokyo, Japan).

### Quantitative polymerase chain reaction (qPCR)

Total RNA was extracted using TRIzol reagent (Invitrogen, CA, U.S.). Reverse transcription of cDNA was carried out using a RevertAid First Strand cDNA Synthesis Kit (Thermo Scientific, CA, U.S.). Gene-specific PCR amplification was performed with Power SYBR Green Master Mix on a Prism 7000 Real-Time PCR System (all from Applied Biosystems, CA, U.S.). Relative gene expression was calculated with the 2^−ΔΔCt^ method after normalization to GAPDH expression. Primers for HIF-1α, vascular cell adhesion molecule-1 (VCAM-1), stromal cell-derived factor-1 (SDF-1), and insulin-like growth factor-1 (IGF-1), are listed in the Supplementary Table S1.

### MicroRNA analysis

An All-in-One miRNA qRT-PCR Detection Kit (GeneCopoeia, MD, U.S.) was used to measure miRNA expression. Briefly, total RNA was treated with poly A polymerase to add poly-A tails to the 3′-end of miRNAs. M-MLV RTase, with a universal adaptor PCR primer, was used to reverse transcribe miRNA tailed poly-A. Then, real-time qPCR was carried out using SYBR Green to measure microRNA expression. RNU6B was used as internal control. Relative expression of each gene was calculated and normalized using the 2^−ΔΔCt^ method. Specific primers for Hsa-miR-155-5p and RNU6B were all from GeneCopoeia. All reactions were run in triplicate.

### *In vitro* microtubule formation assay

Induced ECs or HUVECs (4 × 10^4^) were placed atop 50 mL/well Matrigel (10 mg/mL) in 24-well plates (all from Corning, MA, U.S.). Rearrangement of cells and the formation of capillary-like structures were observed at 6 h. The structures were photographed under a phase-contrast Olympus IX71 microscope. The number of mesh tubules was determined using the image analysis software package ImageJ (http://rsbweb.nih.gov/ij/).

### Construction of plasmids

The 3′-untranslated region (3′-UTR) fragment of the human *E2F2* gene containing miR-155 binding site was amplified by PCR from genomic DNA of HUVECs and then cloned into a pGL3 luciferase reporter gene vector (Promega, WI, U.S.). The E2F2 3′-UTR mutation plasmid was generated by Genewiz (Beijing, China). The coding region of E2F2 was also amplified from HUVECs and inserted into pcDNA 3.1 (Invitrogen, MD, U.S.). The precursor miR-155-5p (pre-155) and its corresponding scramble control cloned into lentiviralvector pEZX-MR04 were generated from GeneCopoeia. The miR-155 inhibitor (inh-155) was synthesized by GeneCopoeia as well. All constructs were verified by sequencing. Specific primers for the E2F2 coding sequence and 3′-UTR with the restriction enzyme cutting site are listed in Supplementary Table S2.

### Luciferase reporter assay

The reporter vector was co-transfected with pre-155 and its scramble control using Lipofectamine 3000 (Invitrogen) according to the protocol provided. pGL3-Luc *Renilla* was used as internal control. Reporter activity was quantified 48 h after transfection using a Dual-Luciferase Reporter Assay Kit (Promega, WI, U.S.). The relative luciferase unit was defined as the ratio of luciferase activity to *Renilla* activity with that of control set as 1.0.

### Western blot analysis

Total protein extracts were obtained by lysing cells in cold RIPA buffer containing a proteinase and a phosphatase inhibitor (Beyotime Biotechnology, Wuhan, China). Cell lysates were separated using 10% SDS-PAGE and transferred to PVDF membranes (Bio-Rad Laboratories, CA, U.S.). Membranes were blocked and immunolabeled overnight at 4 °C with primary antibodies against angiogenic factors VCAM-1, SDF-1 and IGF-1 (all from Santa Cruz Biotechnology, CA, U.S.). Membranes were then incubated with secondary antibodies (Cell Signaling Technology, MA, U.S.). Immunolabeling was visualized using enhanced chemiluminescence (ECL) (Beyotime, Wuhan, China) according to the manufacturer’s instructions.

### Statistical Analysis

All data are representative of 3 independent experiments and expressed as means ± SD. Statistical analysis was carried out using the Student’s t-test. Differences were considered statically significant when *P* < 0.05. Data analyses were performed using GraphPad 5.0 software.

## Additional Information

**How to cite this article**: Yang, D. *et al.* Role of Mir-155 in Controlling HIF-1α Level and Promoting Endothelial Cell Maturation. *Sci. Rep.* **6**, 35316; doi: 10.1038/srep35316 (2016).

## Supplementary Material

Supplementary Table S1

Supplementary Table S2
