# Supplementary material for: Role of Mir-155 in Controlling HIF-1α Level and Promoting Endothelial Cell Maturation
Source: Sci Rep. 2016 Oct 12;6:35316. doi: 10.1038/srep35316 (PMC5059686; doi:10.1038/srep35316)
Supplement: Supplementary Table S1 [file srep35316-s1.doc]

**Role of Mir-155 in Controlling HIF-1α Level and Promoting Endothelial Cell Maturation**

Deguang Yang^1,+^, Jinhong Wang^2,+^, Meng Xiao^3^, Tao Zhou^1,*^, Xu Shi^4,*^

**Table S1.** Primer sequences used in qRT-PCR.

| Gene name | Primer sequence | Ref Seq |
| --- | --- | --- |
| HIF-1α | F: ACCATGCCCCAGATTCAGG  R: AGTGCTTCCATCGGAAGGACT | NM_001530.3 |
| IGF-1 | F: CCATGTCCTCCTCGCATCTC  R: CGTGGCAGAGCTGGTGAAG | NM_001111283.1 |
| SDF-1 | F: TGAGCTACAGATGCCCATGC  R: TTCTCCAGGTACTCCTGAATCC | [NM_199168.3](http://www.ncbi.nlm.nih.gov/nucleotide/291045298?report=genbank&log$=nucltop&blast_rank=4&RID=G8W38CMY014) |
| VCAM-1 | F: ATGACATGCTTGAGCCAGG  R: GTGTCTCCTTCTTTGACACT | [NM_001078.3](http://www.ncbi.nlm.nih.gov/nucleotide/315434269?report=genbank&log$=nucltop&blast_rank=2&RID=G8XZVVKE014) |
| GAPDH | F:GGTGGTCTCCTCTGACTTCAACA  R:GTGGTCGTTGAGGGCAATG | NM_002046 |
