# Supplementary material for: Role of Mir-155 in Controlling HIF-1α Level and Promoting Endothelial Cell Maturation
Source: Sci Rep. 2016 Oct 12;6:35316. doi: 10.1038/srep35316 (PMC5059686; doi:10.1038/srep35316)
Supplement: Supplementary Table S2 [file srep35316-s2.doc]

**Role of Mir-155 in Controlling HIF-1α Level and Promoting Endothelial Cell Maturation**

Deguang Yang^1,+^, Jinhong Wang^2,+^, Meng Xiao^3^, Tao Zhou^1,*^, Xu Shi^4,*^

**Table S2. Primer sequences used in plasmid construction.**

| Gene name | Primer sequence | Ref Seq |
| --- | --- | --- |
| E2F2 3’UTR  With Sac I and Hind III cutting site | F: GCGCGAGCTCCATTGTCCCTTCAGCTCAGC  R: GCGCAAGCTT CCAGGAATTTGAGCAAATG | NM_004091.3 |
| E2F2 CDS  With EcoR I and EcoR V cutting site | F: GCGCGAATTCATGCTGCAAGGGCCCCGGG  R: GCGCGATATCTCAATTAATCAACAGGTCCC |
